# Potential Heavy Metals Pollution Contribution from Wash-Off of Urban Road-Dust

**DOI:** 10.3390/toxics10070397

**Published:** 2022-07-18

**Authors:** Muhammad Faisal, Zening Wu, Huiliang Wang, Xiaoying Lin, Zafar Hussain, Muhammad Imran Azam

**Affiliations:** 1 College of Water Conservancy Engineering, Zhengzhou University, Zhengzhou 450001, China; engineerfaisi@gmail.com (M.F.); zeningwu@zzu.edu.cn (Z.W.); wanghuiliang@zzu.edu.cn (H.W.); zafar775@yahoo.com (Z.H.); 2 Zhengzhou Key Laboratory of Water Resource and Environment, Zhengzhou 450001, China; 3 Water Resources Section, Ministry of Planning, Development & Special Initiatives, Islamabad 44020, Pakistan; 4 Hydropower and Water Resources Section, Zeeruk International (PVT), Islamabad 44020, Pakistan; m.imranazam@hotmail.com

**Keywords:** road-deposited sediments, pollution load, index model, pollution strength, urban diffuse pollution

## Abstract

Based on the different particle sizes of street dust, the potential pollution load of heavy metals from dry and wet atmospheric deposition to surface runoff in different functional areas of Zhengzhou city was estimated by using the rain-scour heavy metal index model. Compared to the EA, IA, and CA areas, RA and PA have a higher potential contribution to heavy metal runoff pollution from Road deposited sediments (RDS) than the other watersheds. Zhengzhou utilized the RDS index model to calculate pollution loads in various areas around Zhengzhou (EA, IA, CA, PA, and RA). In the different land-use areas, the RDS indices for pollutant load (RDS_index,load_) and pollutant strength (RDS_index,strength_) varied greatly, and the RDS _index strength_ values increased. RDS_index,load_ fell in the following order: IA > RA > PA > EA. Because the RDS index incorporates RDS characteristics such as the amount of RDS, grain sizes present, RDS mobility, and associated metals, the RDS_index,load_ and RDS_index,strength_ results did not merely match variability in the amounts of RDS found or metal concentrations in the RDS in various land-use areas. Metal’s presence in the dust is a direct health risk for humans and warrants immediate and effective pollution control and prevention measures in the city.

## 1. Introduction

Heavy metals pose a serious threat to human health, and their increasing presence in urban road dust warrants a health emergency [1,2,3,4]. Surface runoff, which causes diffuse contamination, is a significant contributor to the deterioration of water quality in urban environments [5,6,7]. There have been numerous studies that have revealed significant amounts of heavy metals in soluble form in urbanized water bodies, which has raised the importance of and interest in measuring such loads [8,9,10]. In addition, studies have investigated heavy metals related to the solid fraction of the surface rush, specifically the portion of road-deposited sediments (RDS) that accumulate during dry rainstorm events [11,12]. Road-deposited sediments are a good place to look for contaminants in urban areas because they can move them around [13,14]. The majority of research efforts have concentrated on the direct investigation of heavy metals concentrations associated with road runoff before analyzing the wash-off phenomenon utilizing RDS between rainfall occurrences (which is a relatively new development, e.g., in dry weather). By incorporating dry weather RDS into approaches, it has been possible to simplify previous models of wash-off produced by storm water runoff. As China and many other countries reduce their reliance on point sources of pollution, urban runoff, including contaminated road-deposited sediment (RDS), has become an increasingly critical problem [15,16]. Pollutants are transported by RDS from impermeable surfaces, which frequently contain high concentrations of metals [17,18]. RDS wash-off transports large quantities of contaminants such as fertilizers, metals, and hydrocarbons [19,20]. It is possible that quantifying the link between RDS and wastewater particles in urban runoff will lead to a new method for evaluating the pollution load that a channel receives [21,22]. To predict runoff pollutant loads, various diffuse pollution models have been developed for metropolitan environments, including STORM, DR3M-QUAL, HSPF, SWMM, and SLAMM [23]. Even though the models mentioned above are frequently used to forecast scattered pollution, further study is still required. For characterization and confirmation, urban sources often require a great amount of data [24]. Therefore, more accurate runoff contamination estimates are needed. Critical source regions should be prioritized in order to effectively control RDS wash-off contamination. Because they rely less on input data, index models are frequently employed to locate diffuse critical phosphorus sources on farms [25]. Index models are used to identify important source sites by quantifying the comparative pollution risk rather than the actual pollutant loading because it is difficult to measure transit variables [26]. No index model has yet been created specifically for dispersed pollution in urban areas that may be used to identify important source sites for urban runoff in contrast to the index models outlined above, which were initially established for diffuse pollution in rural regions. It may be possible to locate important urban runoff source sites by taking into account RDS features, such as volume, grain sizes, associated metals, and mobility [27]. A developed risk index called RI_RDS_ takes into account features of the RDS and a possible ecological risk index (RI). It is possible to estimate the amount of pollution in a given area using RI_RDS_ but not its total pollution load. When evaluating the quantity of pollution present per unit area, the RI_RDS_ can be used to determine the overall amount of pollution present in the area. To figure out how much pollution is in the air in cities, new simple but effective index models that use RDS features are needed. Included among the primary goals were (1) assessing the quantity and its grain size distribution of road deposited sediments along the different functional areas and (2) potential pollution contribution of heavy metals in surface runoff, (3) determining the transport and source factors for heavy metals in RDS utilizing weighted and observed RDS features in a multiplicative index, and (4) combining a number of functions for evaluating the pollution strength and load into one RDS index.

## 2. Materials and Methods

### 2.1. Study Area Background

**Figure 1:**
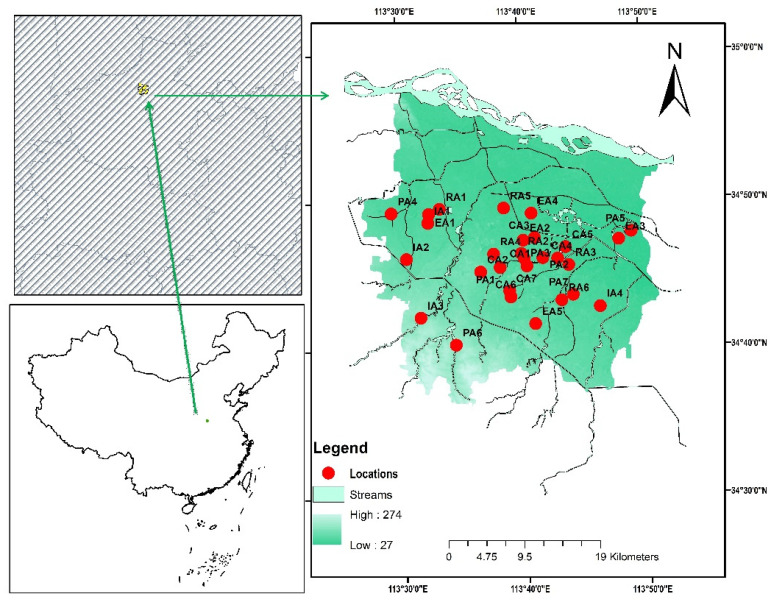
Study Area of Zhengzhou city.

Zhengzhou, the capital of Henan Province, which is located at 34°45′50.4″ N, 113°41′2.4″ E, is depicted in Figure 1. It is an important economic, transportation, and logistical hub in central China. It is the province’s major city, lying in the enormous metropolis of the Central Belt. The city is at the base of the Funiu Hills in the northern part of the country. It is bounded to the west by mountainous terrain and to the east by intermediate and lowland topography. It covers 1011.3 km^2^ of land. The average amount of rain that falls in a year is 629.7 mm, and most of it falls in the summer.

### 2.2. Sampling and Laboratory Analysis

#### 2.2.1. Sample Collection and Rainfall Simulation Experimental Design

For this experiment, 29 RDS sampling sites were selected. Road-deposited sediments (RDS) were collected from a variety of sampling locations around Zhengzhou city; 5 sites from educational (EA), 4 from industrial (IA), 7 from parks (PA), 6 from residential (RA), and 7 from commercial areas (CA). Samples were taken from a different area of the road each time. The central road commemorating the curb was vacuumed first. A ruler was used to measure the size of the sampling area. At each sampling site, a digital weight scale (WANT, model: WT2003, China) was used to weigh the RDS specimen. The weight of the samples collected ranged from 1 to 1.5 kg. With the help of polyester sieves, samples were separated into grain size fractions, with sizes of <40, 40–60, 60–100, 100–150, 150–300, 300–500, and >500 μm. Numerous significant elements influence the potential contribution of RDS to runoff and runoff pollution. It was important to study the RDS particle size and composition as well as rainfall intensity during the RDS runoff experiment. For different particle sizes, it is possible to calculate the RDS percentage contribution by combining the percentages of each particle size. The clearance rate of RDS from surface runoff was estimated using a specifically constructed rainfall simulation and tiny impervious surface plots. Two rainfall simulators comprised the apparatus for simulating rainfall. The swing nozzle boom, which swung out to a height of 2.5 m, had four nozzles each for the two rainfall simulators, spaced evenly apart (by a distance of 1.1 m). An electrical control box controls the rainfall simulators, allowing them to be calibrated for varying rainfall intensities. The nozzles (Veejet 80100) produced medium-sized rain drops at 0.04 Mpa. When used in conjunction, the two rainfall simulators provided a homogeneous rainfall area of 1.5 m × 2.2 m. For our wash-off test plot, we chose an area that was 1.5 m × 2 m. The plot boundary was marked by a 1.5 m × 2 m plastic frame, which was then sealed with a tiled surface. It was necessary to leave one end of the plastic frame open in order to attach the catch tray that was used for runoff collection.

In the wash-off experiment, we assessed the runoff water’s retrieval efficiency by comparing the volume of collected water to the volume of simulated rainfall used in the experiment. The road that was chosen for the RDS wash-off experimental plot had been paved with asphalt a few years prior, and its slope and roughness were 2.37 degrees and 0.624 mm, correspondingly, when measured. Before the wash-off test, the RDS distribution was evenly distributed across the wash-off test plot. Prior to the next simulated rainfall event, we flushed the simulated runoff plot with water pipe to ensure that the plot surface was fully washed. We selected mixed RDS samples from all throughout the study area to ensure we had representative samples for our simulated runoff experiment. Forty-two different rain events with different amounts of rain and three different amounts of RDS mass per square meter were all tested.

#### 2.2.2. Quality Control and Analytical Methods

Five heavy metal elements (Cr, Cu, Ni, Zn, and Pb) in every RDS sample were determined [28]. Hotplate digestion with HF-HclO4 was used to digest RDS samples [29]. The CRMs for RDS digestion are GBW07401 (GSS-1) and GBW07402 (GSS-2). They are certified reference materials (CRMs) for soil, certified by the General Administration of Quality Supervision, Inspection, and Quarantine of the People’s Republic of China. However, soil CRMs have been found to be an appropriate method of assessing the adequacy of RDS analysis in prior research even though no RDS CRMs are available [16,30]. There were detection limits of 2 mg/L for Cr, 0.6 for Cu, 1 for Ni, 1 for Zn, and 2 for Pb. Analyses were performed on 2 percent of the RDS samples that were duplicated; the metal amounts discovered in the duplicates were always within ±10 percent of the mean concentration. Each set of samples was evaluated with a reagent blank included. Samples of runoff water were filtered through Millipore filter paper that had been pre-weighed at 0.45 m. The filter paper was then dried and weighed again to figure out the total suspended solids in the water (TSS). The identical process we used for the filtrate samples was utilized to measure blanks for each batch of samples. Prior to analysis, all solutions were kept at 4 °C. With a confidence level of 95% and a precision of nearly 8%, a Perkin-Elmer Elan 6000 ICP-OES was used to measure Cr, Cu, Ni, Zn, and Pb concentrations.

### 2.3. Heavy Metals Load Estimation

We estimated the pollutant load percentages for every individual RDS sample to quantify the contribution of particles of varying grain sizes to the overall pollution of the RDS. The following is the equation that was used to compute the grain size fraction load (GSF_Load_):

(1) $${\mathrm{GSF}}_{\mathrm{Load}}=\frac{C_{i}{\;\times \;\mathrm{GS}}_{i}}{\sum_{i=1}^{n}C_{i}{\;\times \;\mathrm{GS}}_{i}}\;$$

where C_i_ = heavy metal concentration of sample for a given grain size; GS_i_ = percentage by mass of the size fraction I in the total sample; n = number of grain size fractions.

### 2.4. RDS Percentage in Surface Runoff Estimation

Each simulated rainfall event was followed by the collection of surface runoff samples and the determination of their quantities at predetermined intervals. As a percentage of the overall RDS mass in the runoff from the impermeable surface, each RDS size fraction was reported as a percentage of that mass. This was determined using the following equation:

(2) $$\mathrm{Fw}\;\left(\%\right)=\frac{M_{\mathrm{FW}}}{M_{\mathrm{initial}}}\times 100\%=\frac{\int_{0}^{1}C\left(t\right)\;\times \;Q\left(t\right)\mathrm{dt}}{M_{\mathrm{initial}}}\times 100\%\;$$

where Fw = percentage of each RDS size fraction washed off the surface (%); M_Fw_ = mass of the size faction washed off the surface over the entire rain event (mg); M_initial_ = initial mass of RDS with a corresponding grain size on the surface (mg); C(t) = mass of RDS with a corresponding grain size in the surface runoff water (mg/L) at each sampling time; Q(t) = surface runoff flow rate at each sampling time (m^3^/min).

### 2.5. Surface Runoff Contribution from Heavy Metals

The following equation was used to determine how much pollution could be washed off the surface and into the runoff:

(3) $$\mathrm{Pw}=\overset{7}{\underset{i=1}{\sum }}M_{i}\times C_{i}\times I$$

where Pw = potential pollution contribution to runoff per unit area (μg/m^2^); M_i_ = mass of RDS of one size fraction per unit area (g/m^2^); C_i_ = mass of heavy metal in each size fraction of RDS (mg/kg); Fw_i_ = percentage of each RDS size fraction washed off the surface (%).

### 2.6. Metals Source and Transport Factors in Road-Deposited Sediments

#### 2.6.1. Source Factors

When it comes to surface runoff, the amount of pollutants contained in RDS is critical since it determines the amount of pollutants present in surface runoff. The RDS index models used source factors such as RDS levels, grain sizes, and contaminants linked with RDS. We looked at a variety of functional areas and a large number of sites to obtain the best RDS parameter estimates possible. There was a large range in the mass of RDS per unit area, with a mean value of 70 g/m^2^. Each RDS grain size fraction was analyzed for metal concentrations.

#### 2.6.2. Transport Factors

Simulated rainfall was used to assess the particle mobility in each RDS grain size fraction. Surface runoff was estimated using a specifically constructed rainfall simulator and impermeable plots of land. Equation (2) was used to compute the mass of RDS in the impervious surface runoff as a percentage of the overall mass of RDS in the runoff compared to the starting mass of RDS on the surface. In order to produce Fw, 42 different simulated wash-off scenarios were run for 1 h each with seven RDS size fractions (<40, 40–60, 60–100, 100–150, 150–300, 300–500, >500 μm), and simulated rainfall intensities ranged from (10 to 120.3 mm/h). Fw was used to determine the amounts of RDS washed off for various particle sizes as well as the levels of heavy metals connected with the washed-off particles. Despite the fact that the bulk RDS particles’ Fw was influenced by their grain size distribution, the Fw for individual grain size particles was unaffected [22,31]. In order to accurately depict the RDS wash-off scenarios in our calculations, we used the wash-off percentage for each RDS grain size to compute the bulk RDS wash-off percentage and amount for the relevant sampling region. We calculated the amount of RDS wash-off that happened for the RDS samples taken from each functional region using the Fwi values for each RDS size fraction.

#### 2.6.3. Factor Ratings for Source and Transport

According to our in situ observations of the RDS features, we allocated weightings to the source and transport elements. The RDS mass per square meter was ranked into six groups and is presented in Table 1 [19,27];

The transport variables can be graded according to the percentages of RDS washed-off grains in each grain size fraction (Fwi). The transport factors were included into the RDS index by assigning a value of 1 to the Fw value, with the lowest value for a particular grain size fraction (Table 2). The lowest Fw value was used to determine the Fw values for different grain size fractions.

### 2.7. RDS Index Calculation

This section will explain the fundamental concepts underlying the RDS index calculation. The RDS index is based on the phosphorus index, which is used to model diffuse pollution in rural areas, but it was specifically built to deal with the RDS features seen in diffuse pollution in urban areas [25,32]. The RDS index is utilized in this study to measure real pollutant loadings as well as identify critical source sites by calculating relative pollution risks. It is important to remember that source and transport factors are taken into consideration in both quantitative and semi-quantitative applications that use RDS features. The weighted RDS characteristics and the measured RDS characteristics were used to determine semi-quantitative and quantitative pollutant loadings, respectively. Equation (4) was used to derive the RDS index.

(4) $${\mathrm{RDS}}_{\mathrm{index}}=F_{\mathrm{source}}\times F_{\mathrm{transport}}$$

where F_source_ = source factor, and F_transport_ = transport factor.

#### 2.7.1. Index Model for Load

For this particular load, we computed the RDS index model based on the observed source and transport factor values using Equation (7). F_source,load_ and F_transport,load_ were determined independently utilizing Equations (5) and (6) as the inputs to the load calculations.

(5) $$F_{\mathrm{source},\mathrm{load}}=\overset{m}{\underset{j}{\sum }}\overset{n}{\underset{i}{\sum }}\left(M_{i}\times C_{\mathrm{ij}}\times A\right)\;$$

(6) $$F_{\mathrm{transport},\mathrm{load}}=F_{w,\mathrm{observed}\;}$$

(7) $$\begin{array}{ll} {\mathrm{RDS}}_{\mathrm{index},\mathrm{load}} & =F_{\mathrm{source},\mathrm{load}}\times F_{\mathrm{transport},\mathrm{load}}\\ & =\overset{m}{\underset{j}{\sum }}\overset{n}{\underset{i}{\sum }}\left(M_{i}\times C_{\mathrm{ij}}\times A\times F_{\mathrm{wi},\mathrm{observed}}\right) \end{array}$$

where RDS_index,load_ = potential amount of pollution in the runoff (kg); Mi = mass of a particular RDS size fraction per unit area (mg/m^2^); Cij = measured concentration of the metal of interest j in the RDS with a grain size i (mg/kg); i and j = numbers of grain size fractions and metal species in the study, respectively; A = road surface area.

#### 2.7.2. Index Model for Pollution Strength

Equation (10) was used to generate the RDS index for pollution strength based on the weighted values of the source and transport factors. Equations (8) and (9) were used to calculate the F_source,strength_ and F_transport,strength_.

(8) $$F_{\mathrm{source},\mathrm{strength}}=\overset{m}{\underset{j}{\sum }}\overset{n}{\underset{i}{\sum }}\left(T_{r}^{j}\times \frac{C_{\mathrm{ij}}}{C_{\mathrm{rj}}}\times \mathrm{Pi}\times M_{\mathrm{weighted}}\right)\;$$

(9) $${\;F}_{\mathrm{transport},\mathrm{strength}}=F_{w,\mathrm{weighted}}$$

(10) $$\begin{array}{ll} {\mathrm{RDS}}_{\mathrm{index},\mathrm{strength}} & =F_{\mathrm{source},\mathrm{strength}}\times F_{\mathrm{transport},\mathrm{strength}}\\ & =\overset{m}{\underset{j}{\sum }}\overset{n}{\underset{i}{\sum }}\left(T_{r}^{j}\times \frac{C_{\mathrm{ij}}}{C_{\mathrm{rj}}}\times \mathrm{Pi}\times M_{\mathrm{weighted}}\times F_{\mathrm{wi},\mathrm{weighted}}\right) \end{array}$$

where RDS_index,strength_ = potential pollution strength in the runoff; $T_{r}^{j}$ = toxic response factor, and the values were Cr = 2, Cu = 5, Zn = 1, Ni = 3, and Pb =5 [33]. Pi = amount of RDS, with grain size i = percentage of the total RDS mass; M_weighted_ = level of the bulk RDS mass per unit area for each sampling site.

The RDS_index,strength_ method was assigned values and categories based on the products of the source and transit factors. RDS_index,strength_ was classified as RDS_index,strength_ ≤ 150 (low risk), 150 < RDS_index,strength_ ≤ 300 (moderate risk), 300 < RDS_index,strength_ ≤ 600 (considerable risk), and RDS_index,strength_ > 600 (high risk) [27].

## 3. Results and Discussions

### 3.1. Concentration of Heavy Metals in RDS

**Figure 2:**
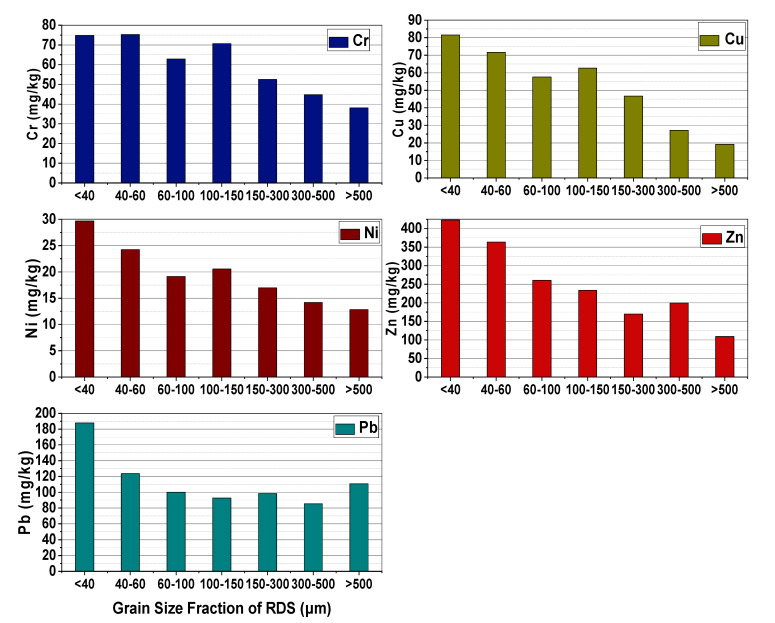
Heavy metal concentrations in RDS.

In the occurrence of heavy metals, this study looked at five different metals (Cr, Cu, Ni, Zn, and Pb) in each size group of the RDS (Figure 2), and background values of these heavy metals in Zhengzhou were 64, 14, 21, 42, and 18, respectively [34].

In these metals, Zn has the highest concentration in the grain size fractions of <40, 40–60, 60–100, 100–150, 150–300, and 300–500 µm, while Ni has the lowest concentration, but for the grain size fraction of >500 µm, Pb has the highest concentration. Most heavy metal concentrations were substantially greater than their respective background values. The highest metal concentrations were found in the tiniest RDS particles <40 µm for all the metals except for Cr, where the highest concentration found in 40–60 µm size fraction. However, the lowest concentrations of all the heavy metals were found in the grain size of >500 µm except for Pb, which has the lowest concentration value in the grain size fraction of 300–500 µm. By and large, the fractions with smaller particle sizes had more heavy metals in them.

Due to the fact that this research was conducted in five distinct functional areas, Table 3 summarizes the concentrations of the five heavy metals (Cr, Cu, Ni, Zn, and Pb) by functional area and grain size distribution. To put it another way, RA had the highest concentrations of Cr and Ni, while EA had the lowest concentrations of Cr and Ni. Unexpectedly, the IA had the lowest quantities of Cu, Zn, and Pb, whereas the PA had the greatest concentrations of Zn and Pb, and the CA had the highest concentrations of Cu.

### 3.2. Heavy Metal Loads of RDS

**Figure 3:**
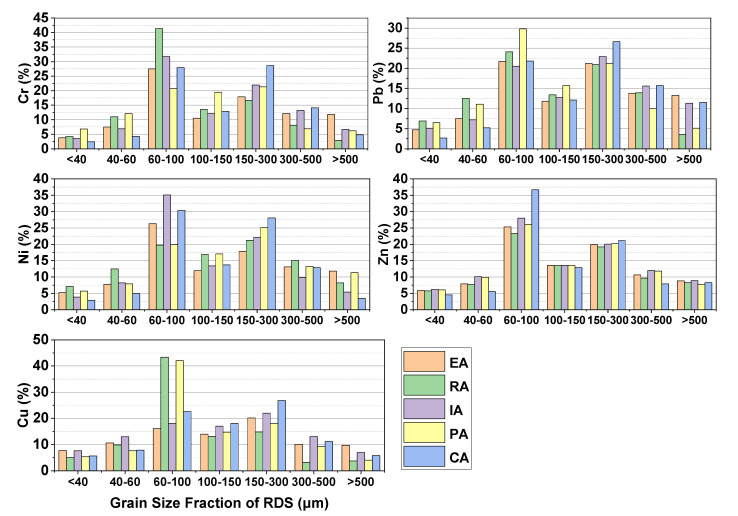
GSF_Load_ (%) of different grain size fractions (μm) for different functional areas.

GSF_Load_ was used to calculate the percentage of each RDS size fraction’s heavy metal load that was contributed by that size fraction. The percentage contributions of mean GSF_Load_ for each metal were as follows: 60–100 μm > 15–300 μm > 100–150 μm > 300–500 μm > 40–60 μm > 500–1000 μm > 0–40 μm (Figure 3). By and large, heavy metals were concentrated in particles with a diameter of < 3000 μm in the RDS. It is an usual practice to refer to RDS with a grain size of less than <100 μm as the grain size of suspended particles in runoff [35]. Because of this, the contribution of particles with an average diameter of <100 μm to the overall metal contamination of the RDS has an impact on the possible emission of pollutants to runoff pollution. When all metals were considered together, the percentage contributions of the <100 μm particles in the various functional areas to the mean GSF_Load_ (%) were in the following order: RA > PA > IA > EA > CA.

### 3.3. Surface Runoff RDS and Heavy Metal Loads Estimation from Simulated Rainfall

To quantify the contribution of RDS to urban runoff, it is necessary to have a thorough understanding of how the various RDS size fractions are washed off impervious surfaces. Rainfall simulations were used to figure out how much RDS (Fw) washed away from the impervious surface for each grain size fraction. At three different levels of RDS mass per unit area, these percentages were found (Table 4). In this case, Fw was related to the amount of rain that fell at a given spatial density of particles on the surface. It was not related to the spatial density of particles [36]. RDS was rapidly rinsed from the pavement due to its tiny size. The initial flush happened, and it was more noticeable with tiny particles than with bigger particles.

**Figure 4:**
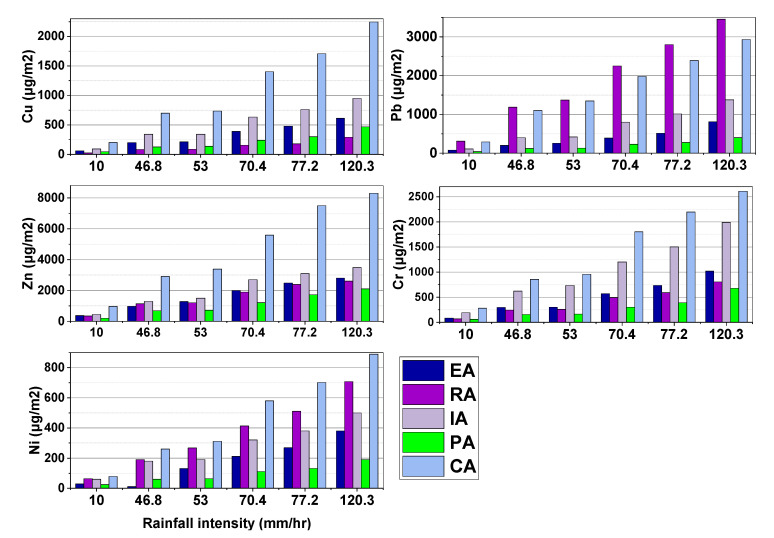
Potential pollution contributions to runoff per unit area (μg/m^2^).

Using RDS washed off impermeable surfaces, Figure 4 depicts the possible contribution to surface runoff pollution of washed-off RDS. For Cr and Ni at (10–120.3 mm/h) and Zn and Pb at (10–53 mm/h), the areas for possible pollution contribution per unit area of impervious surface were as follows: CA > IA > EA > RA > PA. CA > IA > RA > EA > PA was the order for Cu (10–120.3 mm/h) and Pb and Zn (53–120.3 mm/h). The disparity between RDS and the metals linked to it in terms of potential pollution contribution per unit area grows higher as rainfall intensity increases. This could be explained by the fact that smaller particles have higher mobility and GSF_Load_ than larger ones.

Rainfall intensity has a significant effect on the potential pollutant contribution per unit area in different functional sectors. The more intense the rain, the more the contribution went up. Compared to the EA, IA, and CA areas, RA and PA have a higher potential contribution to heavy metal runoff pollution from RDS than the other watersheds. Pollution from heavy metals could be higher in RA and PA because there were much smaller particles in RDS, which had more metal in them and were easier to wash away in runoff.

### 3.4. Source and Transport Factors in Road-Deposited Sediments (RDS) Index

**Figure 5:**
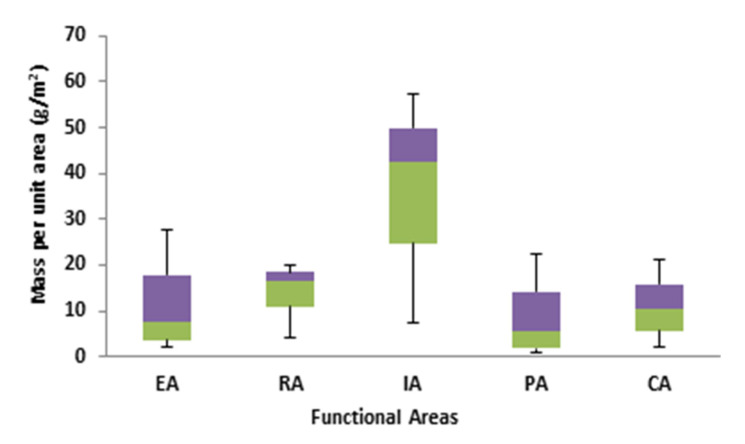
Box plot of the amount of RDS in functional areas.

The source elements in the RDS index used in this study comprised the number and distribution of RDS particles of different grain sizes as well as the presence of metals related to the RDS particle distribution. In the city, five distinct functional regions (EA, RA, PA, IA, and CA) were explored for source factors. The mass of RDS per unit area notably differed between functional regions, with the RA 25.1 g/m^2^ and PA 23.2 g/m^2^ having the lowest mass per unit area, the EA 55.2 g/m^2^ having the medium mass per unit area, and the CA 138.7 g/m^2^ and IA 185.5 g/m^2^ having the highest mass per unit area as shown in (Figure 5).

**Figure 6:**
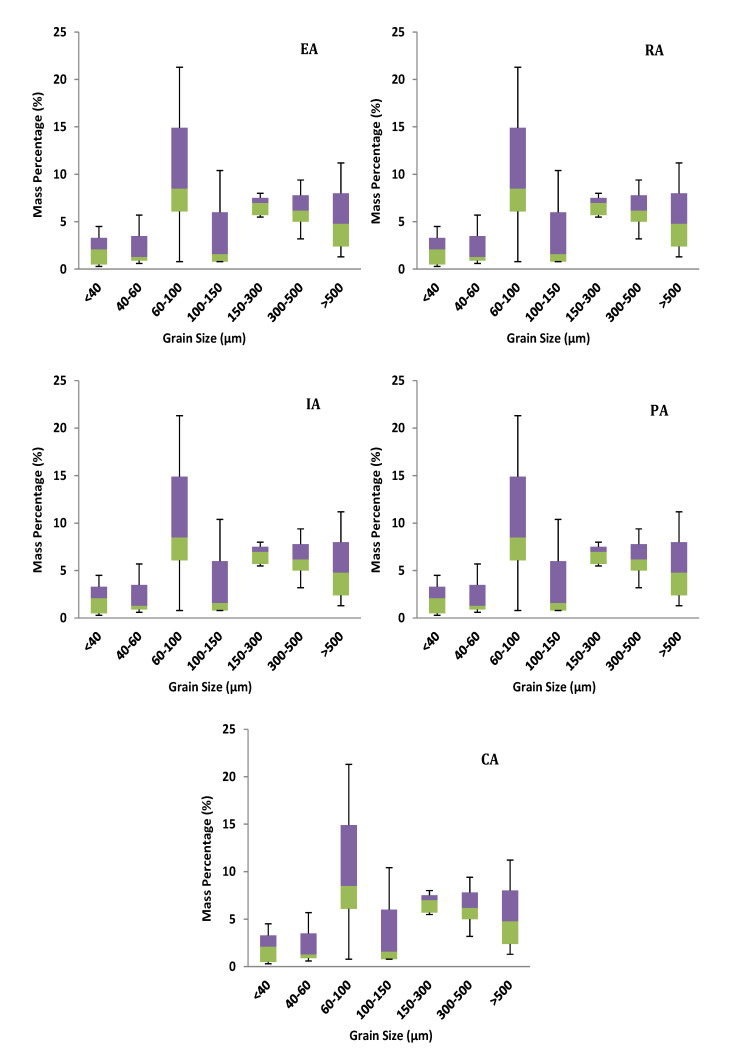
Box plots of the mass percentages of RDS particles in each grain size.

There were significant variations in the RDS particle size distribution across various functional locations (Figure 6). Our site inspections revealed that the IA and CA had large amounts of bare soil or cracked and rough road surfaces, indicating that they were contaminated. While brooms are frequently used to sweep the streets in the IA and CA, in metropolitan areas, motorized sweepers, which have greater RDS removal efficiency, are employed.

**Figure 7:**
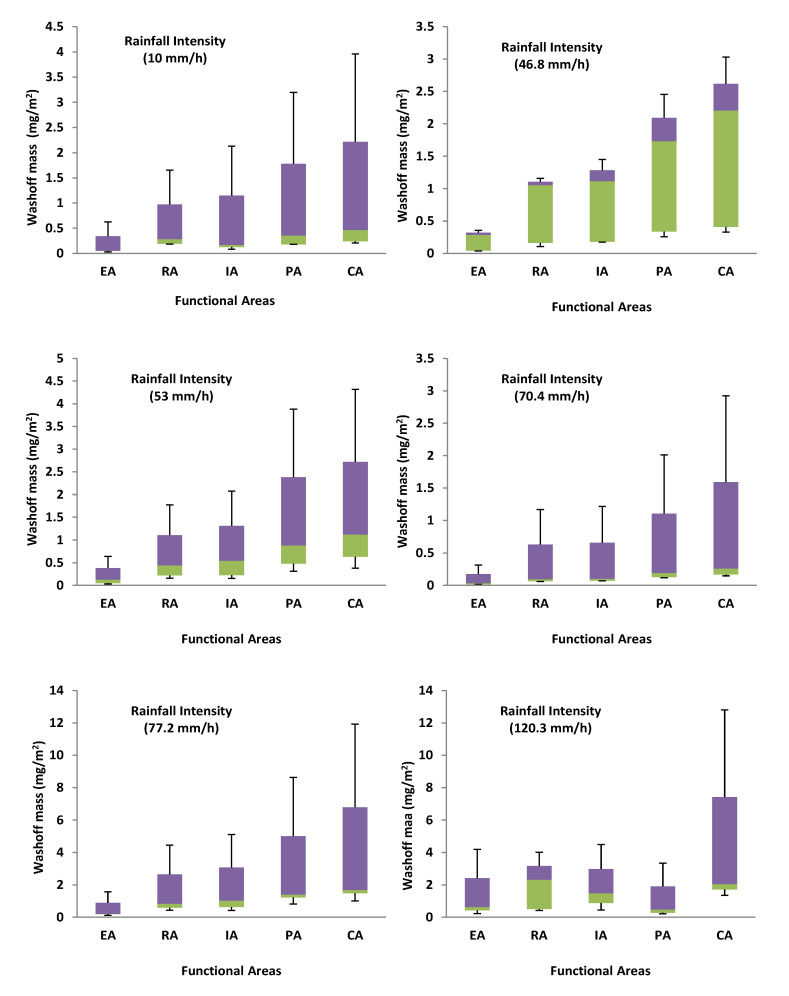
Box plots of the metal concentrations in the RDS in different functional areas.

The transport factors investigated in this research were chosen primarily for their ability to alter surface runoff, which in turn affects the RDS index. The transport variables in the RDS index were represented by the proportion of each RDS particle size washed off the surface (Fwi), and the Fwi observed values ranged from 0.99% to 77.21%. Using the Fwi observed values, the amount of wash-off was determined for each of the RDS size fractions by changing the quantities and particle size compositions in the RDS to correspond to those in the sampling areas [24]. Figure 7 illustrates the RDS wash-off quantities along the functional areas. Increases in rainfall intensity increased the number of particles washed off per unit area, but wash-off as a percentage of RDS was reduced throughout all the functional areas.

### 3.5. Road-Deposited Sediments (RDS) Index for the Pollutant Load

The RDS index for pollutant load estimates pollutant loads in large-scale urban runoff in a novel way. The RDS mobility, amount, and related metals as well as each functional region were combined to create the RDS index for the pollutant load. One hour of simulated rainfall was used, and surface runoff samples were taken manually until no further runoff occurred. From 29.9–910 kg of RDS index, load values were found in the six rainfall intensities studied in the following order: IA > RA > CA > PA > EA (Table 5). As a result of this study’s analysis of RDS features (the amount of RDS in each region, its mobility, and metal concentrations), it may be determined which regions have the greatest potential for pollution due to surface runoff. The amount of pollution carried by urban runoff rose as the severity of the rainfall increased, indicating that dispersed pollution generated by RDS runoff during larger rainfall events should be given particular consideration.

### 3.6. Road-Deposited Sediments (RDS) Index for the Pollutant Strength

**Figure 8:**
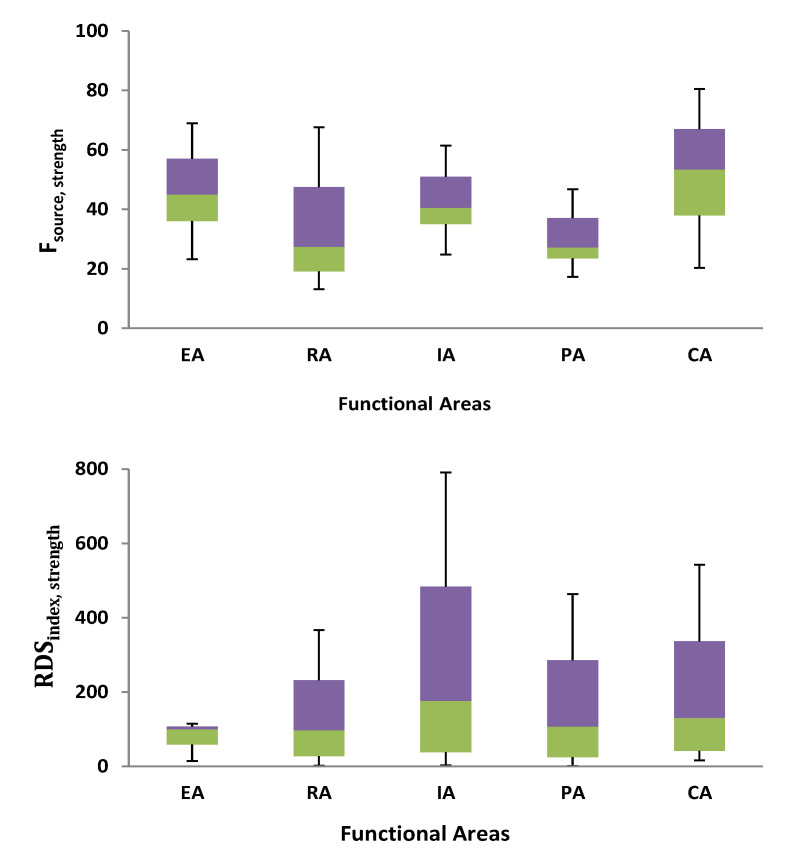
Box plots of the source factors and RDS index for pollution strength.

New pollutant strength RDS indexes for large-scale urban runoff have been developed in a similar way to the RDS indexes for pollutant load. The pollutant strength RDS index incorporated the RDS mobility, quantity, grain size distribution, related metal concentration, and cytotoxicity of the metals into a single measure of pollutant strength. The RDS_index,strength_ (Figure 8) values were in the following order: IA > CA > PA > RA > EA. The order of RDS_index,strength_ is marginally more diverse than F_source,strength_. When it came to establishing the order of the RDS_index,strength_ values, F_source,strength_ was more relevant than F_transport, strength_. To put it another way, the median RDS_index,strength_ value for metals in the IA RDS fell into the considerable risk category, while the other values were all moderate risk. Our findings contribute to a better knowledge of the dangers linked with metal contaminants in urban water that cause RDS.

## 4. Conclusions

Both RDS and runoff samples were analyzed for the presence of five heavy metals (Cr, Cu, Ni, Zn, and Pb). The RDS mass per unit area reduced in the following order: CA (145 g/m^2^) > IA (139 g/m^2^) > EA (63 g/m^2^) > PA (25 g/m^2^) > RA (23 g/m^2^). Zn has the highest concentration in these metals in the grain size fraction of < 40, 40–60, 60–100, 100–150, 150–300, and 300–500 µm, while Ni has the lowest concentration, but for the grain size fraction of >500 µm, Pb has the highest concentration. Mostly heavy metals concentrations were substantially greater than their respective background values. In the RA and PA, the RDS had more metal concentrations and more small particles, but in the IA and CA, the RDS had more metals per unit area. RA had the highest concentrations of Cr and Ni, while EA had the lowest concentrations of Cr and Ni. Unpredictably, the IA had the lowest quantities of Cu, Zn, and Pb, whereas the PA had the greatest concentrations of Zn and Pb, and the CA had the highest concentrations of Cu. Controlling the initial flush in the RA and PA as well as enhancing existing street cleaning procedures and road surface conditions in the EA, CA, and IA will be effective measures for reducing RDS runoff pollution according to our findings. The percentage contributions of mean GSF_Load_ for each metal were as follows: 60–100 μm > 15–300 μm > 100–150 μm > 300–500 μm > 40–60 μm > 500–1000 μm > 0–40 μm. When all metals were considered together, the percentage contributions of the <100 μm particles in the various functional areas to the mean GSF_Load_ (%) were in the following order: RA > PA > IA > EA > CA. Rainfall intensity has a significant effect on the potential pollutant contribution per unit area in different functional sectors. The more intense the rain, the more the contribution went up. Compared to the EA, IA, and CA areas, RA and PA have a higher potential contribution to heavy metal runoff pollution from RDS than did the other watersheds. We enhanced an RDS index model and utilized it to calculate pollution loads in various areas around Zhengzhou (EA, IA, CA, PA, and RA). In the different functional sectors, the RDS indices for pollutant load (RDS_index,load_) and pollutant strength (RDS_index,strength_) varied greatly, and the RDS_index,strength_ values increased. For the primary roads, the RDS_index,load_ fell in the following order: IA > RA > PA > EA. Because the RDS index incorporates RDS characteristics such as the amount of RDS, grain sizes present, RDS mobility, and associated metals, the RDS_index,load_ and RDS_index,strength_ results did not merely match variability in the amounts of RDS found or metal concentrations in the RDS in various functional areas. The RDS index is novel a technique for analyzing pollution caused by RDS wash-off in order to aid in its management and control.

## Figures and Tables

**Table 1:** RDS mass per unit area ranking levels.

**M_weighted_**	0–30 g/m^2^	31–60 g/m^2^	61–90 g/m^2^	91–140 g/m^2^	141–190 g/m^2^	>190 g/m^2^
**Ratings**	1	1.75	2.5	3	3.5	3.75

**Table 2:** Ratings of transport factors in RDS index.

Transport Factor	RDS Grain Size Fraction (µm)						
	<40	40–60	60–100	100–150	150–300	300–500	>500
**Fw_i, weighted_**	17	10	4.5	4.3	2.9	1.5	1

**Table 3:** Concentrations of heavy metals (mg/kg) in different grain size fractions for different functional areas.

Heavy Metals	Functional Areas	Grain Size Fraction of RDS (μm)						
		<40	40–60	60–100	100–150	150–300	300–500	>500
Cr	EA	48.47	48.76	40.71	45.72	33.99	28.96	24.63
	RA	106.96	107.6	89.84	100.9	75.01	63.92	54.36
	IA	55.35	55.68	46.49	52.21	38.82	33.07	28.13
	PA	74.94	75.4	62.95	70.7	52.56	44.78	38.09
	CA	89.9	90.45	75.52	84.81	63.05	53.72	45.7
Cu	EA	57.67	50.64	40.68	44.29	32.97	19.15	13.5
	RA	108.62	95.37	76.62	83.43	62.11	36.07	25.43
	IA	51.52	45.24	36.34	39.57	29.46	17.11	12.06
	PA	56.92	49.98	40.15	43.72	32.55	18.9	13.32
	CA	112.01	98.34	79.01	86.03	64.04	37.19	26.22
Ni	EA	19.86	16.21	12.8	13.75	11.34	9.47	8.58
	RA	46.21	37.71	29.78	31.99	26.38	22.04	19.96
	IA	13.67	11.16	8.81	9.46	7.8	6.52	5.91
	PA	22.63	18.47	14.58	15.67	12.92	10.79	9.78
	CA	34.77	28.37	22.4	24.07	19.85	16.58	15.02
Zn	EA	332.28	285.74	204.63	183.58	133.17	156.44	85.61
	RA	542.34	466.37	334	299.64	217.36	255.34	139.74
	IA	132.24	113.72	81.44	73.06	53	62.26	34.07
	PA	671.5	577.44	413.54	371	269.12	316.15	173.02
	CA	371.93	320.52	229.54	205.93	149.38	175.48	96.03
Pb	EA	76.63	50.39	40.83	37.8	40.11	34.87	45.18
	RA	184.77	121.49	98.46	91.15	96.71	84.09	108.94
	IA	41.45	27.25	22.09	20.45	21.69	18.86	24.44
	PA	461.42	303.39	245.88	227.64	241.52	210	272.06
	CA	143.29	94.21	76.35	70.69	75	65.21	84.49

**Table 4:** The wash-off (%) of RDS on different grain size fraction at different levels of RDS by rainfall simulation.

RDS Mass	Rainfall Intensity	Rainfall Duration	Grain Size Fraction of RDS (μm)						
			<40	40–60	60–100	100–150	150–300	300–500	>500
10 (g/m^2^)	10.0 (mm/h)	0–60 (min)	17.16	9.87	4.26	3.98	2.84	1.35	0.99
10 (g/m^2^)	46.8 (mm/h)	0–60 (min)	42.71	28.51	24.59	6.13	4.55	2.58	2.32
20 (g/m^2^)	53.0 (mm/h)	0–60 (min)	52.65	39.94	29.06	7.86	7.08	4.09	3.46
20 (g/m^2^)	70.4 (mm/h)	0–60 (min)	54.68	46.82	46.74	16.61	9.25	5.39	4.12
50 (g/m^2^)	77.2 (mm/h)	0–60 (min)	65.59	70.45	53.01	31.89	10.4	5.55	4.79
50 (g/m^2^)	120.3 (mm/h)	0–60 (min)	77.21	63.8	43.54	39.84	16.39	6.15	3.98

**Table 5:** Combined load (RDS_index,load_) in (kg) of heavy metals in all functional areas.

Functional Areas	Rainfall Intensity (mm/h)					
	10	46.8	53	70.4	77.2	120.3
EA	29.9	107	119.2	190.3	247.1	300.1
RA	51.2	200.1	189.6	335.9	434.1	609.3
IA	98.3	372	402.1	701.3	790.7	910
PA	41.3	140.2	162.3	268.5	322.9	367.5
CA	50.3	250.3	333.9	389.1	403.2	578

## Data Availability

Laboratory results of this framework are collected from the physical sampling of the study area.
